# Assessing Immunization Coverage and the Negative Impact of Local Vaccine Production Cessation in Ecuador

**DOI:** 10.3390/vaccines13040348

**Published:** 2025-03-25

**Authors:** Esteban Ortiz-Prado, Lissette Carolina Villacreses-Brito, Jorge Vasconez-Gonzalez, Cristina Anabel Jacome, Marlon Arias-Intriago, Juan S. Izquierdo-Condoy

**Affiliations:** 1 One Health Research Group, Faculty of Health Science, Universidad de Las Americas, Quito 170604, Ecuador; l.carolina9611@gmail.com (L.C.V.-B.); jorgeedvasconez@gmail.com (J.V.-G.); marlon.6.arias@gmail.com (M.A.-I.); juan1izquierdo11@gmail.com (J.S.I.-C.); 2 Dirección Nacional de Inmunización, Ministerio de Salud del Ecuador, Quito 170604, Ecuador; chrisjacomeo@gmail.com

**Keywords:** vaccine coverage, local production, immunization, public health, Ecuador, BCG, DTP

## Abstract

Background: The COVID-19 pandemic highlighted vaccine importance while exposing inequities in global immunization, especially in LMICs like Ecuador. Local vaccine production ensures supply, reduces reliance on imports, and boosts health security. Understanding the relationship between local production and vaccination outcomes is crucial to addressing emerging public health challenges. Objective: The objective was to assess the impact of local vaccine production cessation on vaccination coverage rates for BCG- and DTP-containing vaccines. Methodology: This retrospective cross-sectional study analyzed vaccine coverage data from 2004 to 2023, focusing on key vaccines such as BCG and DTP, to assess the impact of the cessation of local vaccine production. Mann–Whitney U tests were conducted to compare vaccination coverage during the periods of local production (2004–2013) and post-production cessation (2014–2023). Historical context and policy implications were also evaluated to provide a comprehensive perspective. Results: A significant decline in vaccine coverage was observed following the cessation of local production. For BCG, median coverage decreased from 100% during the production period to 87.8% post-cessation (*p* < 0.0001). Similarly, DTP coverage dropped from a median of 99.5% to 83.4% (*p* < 0.0001). The findings highlight the critical role of local production in maintaining high vaccination rates and ensuring immunization equity. Conclusions: Reinvesting in local vaccine production is pivotal to improving immunization outcomes and strengthening Ecuador’s health security. International collaboration and strategic planning can help overcome current challenges, positioning Ecuador as a regional leader in vaccine production and public health resilience.

## 1. Introduction

Since the creation of the Expanded Programme on Immunization (EPI) in 1974 by the World Health Organization (WHO), vaccines have played a fundamental role in achieving universal coverage against common childhood diseases [1]. Countries joined forces to combat illnesses such as diphtheria, tetanus, pertussis, polio, measles, and tuberculosis. Since its implementation, the program has yielded significant results, reducing mortality and the burden of multiple preventable diseases.

Local vaccine production is essential to meeting a significant portion of local demand. Since the inception of vaccination campaigns, countries have strived to produce their own vaccines, import those they cannot manufacture, and thus maintain a supply that aligns with local production capacities [2]. This local manufacturing not only ensures continued immunization under normal circumstances but also becomes critical during global crises, such as wars, trade border closures, geopolitical conflicts, or worldwide quarantines. In such situations, domestic production helps mitigate shortages and sustain vaccination efforts, preventing disruptions in immunization programs even in the face of global turmoil [3].

Local vaccine production provides multiple advantages. It ensures a reliable and timely supply of vaccines, reduces dependency on international manufacturers, and enhances a country’s ability to respond quickly to public health emergencies, such as pandemics [2,4]. Beyond health security, local manufacturing contributes to economic growth, technological innovation, and equitable vaccine access [5,6]. Notable successes in countries like Thailand demonstrate that capacity building for vaccine production can transform immunization programs and pandemic preparedness [7].

The COVID-19 pandemic underscored the critical role of vaccines in protecting public health while exposing vulnerabilities in global immunization systems [8]. These challenges are particularly pronounced in low- and middle-income countries (LMICs) like Ecuador, where disparities in vaccine access and equity hinder effective disease prevention [9]. The COVID-19 pandemic has significantly impacted vaccine coverage, driven by multiple factors, including increased vaccine hesitancy, disruptions in supply chains, and weakened epidemiological surveillance systems [10]. For instance in May 2020 alone, 105 out of 183 campaigns were postponed or canceled in 57 countries due to COVID-19, with an estimated 796 million vaccine doses postponed or not administered [11,12]. The suspension of vaccination services due to the pandemic put at least 80 million children under one year of age at risk [13]. It has been observed that during the pandemic, vaccines delivered to infants fell by nearly a quarter, specifically 28.9%; deliveries of MMR vaccines dropped by 50.8%; and the human papillomavirus (HPV) vaccine fell by 78.1%. As for the BCG vaccine, there was a 25% reduction in global coverage [13,14].

In this context, Ecuador has undergone multiple phases in its history regarding local vaccine production, demonstrating its potential for achieving self-sufficiency in immunization efforts. For decades, the country successfully produced essential vaccines, such as Bacille Calmette–Guérin (BCG), diphtheria–tetanus–pertussis (DTP), and pentavalent vaccines, significantly contributing to public health [15]. This local production was sufficient to meet domestic demand. However, the cessation of vaccine production in 2013–2014 marked a turning point for Ecuador’s biotechnological industry.

Historically, Ecuador’s vaccine production was led by the Instituto de Higiene y Medicina Tropical Izquieta Pérez. However, political decisions during the 2006–2016 administration led to the discontinuation of local vaccine manufacturing. During this period, substantial revenues from oil exports fueled modernization initiatives, including ambitious plans to revamp vaccine and antivenom production. Bureaucrats at the time assessed that the existing plants did not fully comply with Good Manufacturing Practices (GMPs) and decided to build a new facility instead [16].

Despite this decision, the project never materialized as intended. The government acquired land, purchased equipment—much of which was never used—and ultimately ceased procuring vaccines produced by its own national institute. As a result, local vaccine production came to a permanent halt, and the envisioned new plant was never constructed. Consequently, Ecuador now imports all vaccines and even antivenoms, relying entirely on external suppliers.

This abrupt shift away from local production appears to correlate with a decline in vaccination coverage in the following years, a trend that persisted and worsened during the COVID-19 pandemic. The case of Ecuador exemplifies how the loss of domestic vaccine production capacity can have long-term consequences for public health resilience, making it imperative to reconsider strategies for self-sufficiency in immunization programs.

Declining vaccination coverage remains a pressing public health issue, with direct implications for the resurgence of preventable diseases. Addressing this problem requires innovative, sustainable solutions, including the establishment and support of local vaccine production. The objective of this study was to evaluate the impact that the cessation of local vaccine production has had on vaccination coverage rates for vaccines containing BCG and DTP in Ecuador.

## 2. Materials and Methods

### 2.1. Study Design

This retrospective cross-sectional study analyzed vaccination coverage data in Ecuador over two distinct periods: the Local Production Period (2003–2013), during which vaccines were domestically produced, and the Post-Production Cessation Period (2014–2023), after local production ceased. The objective was to assess the impact of local vaccine production cessation on vaccination coverage rates for BCG- and DTP-containing vaccines.

### 2.2. Data Sources and Variables

This study utilized official records from Ecuador’s key health institutions, including the Instituto Nacional de Higiene y Medicina Tropical Leopoldo Izquieta Pérez (INHMT-LIP), which managed vaccine production during the Local Production Period, and the public pharmaceutical company Enfarma EP, which oversaw vaccine production until its closure. Data on vaccine acquisitions post-2014 were obtained from the Ministry of Public Health (MSP). Additionally, records from the Pan American Health Organization (PAHO) and the World Health Organization (WHO) were used to contextualize and compare regional vaccination trends. The analysis focused on two primary variables: vaccination coverage and local production/acquisitions. Annual vaccination coverage percentages for BCG- and DTP-containing vaccines from 2003 to 2023 were segmented into two periods: the Local Production Period (2003–2013), when domestic production was active, and the Post-Production Cessation Period (2014–2023), when vaccine reliance shifted to imports. Coverage rates were sourced from official reports by the MSP, PAHO, and WHO. Historical production data were derived from INHMT-LIP and Enfarma EP records, while post-2014 data on imported vaccine quantities and costs were provided by the MSP.

### 2.3. Statistical Analysis

To evaluate the differences in vaccination coverage between the two periods, a Mann–Whitney U test was conducted for both BCG- and DTP-containing vaccines. This non-parametric test was chosen due to the non-normal distribution of the data. Dependent variables included annual vaccination coverage rates for BCG and DTP. Independent variables included vaccine production status (local production active or ceased). A violin plot was used to represent the comparison of BCG and DTP vaccine coverage during the local production period and the production cessation period.

Descriptive statistics, including medians and interquartile ranges, were calculated for both periods. The Hodges–Lehmann estimate was used to quantify the median difference between the two periods. Temporal trends in vaccination coverage were visualized using a line graph.

Additionally, a financial allocation analysis for vaccines in Ecuador during the 2013 period was also conducted, and, finally, a timeline was created to summarize the history of vaccine production in Ecuador.

### 2.4. Ethical Considerations

This study was based entirely on secondary data obtained from official institutions, ensuring confidentiality and compliance with ethical standards for research.

## 3. Results

### 3.1. Specific Vaccine Coverage Trends

**Figure 1:**
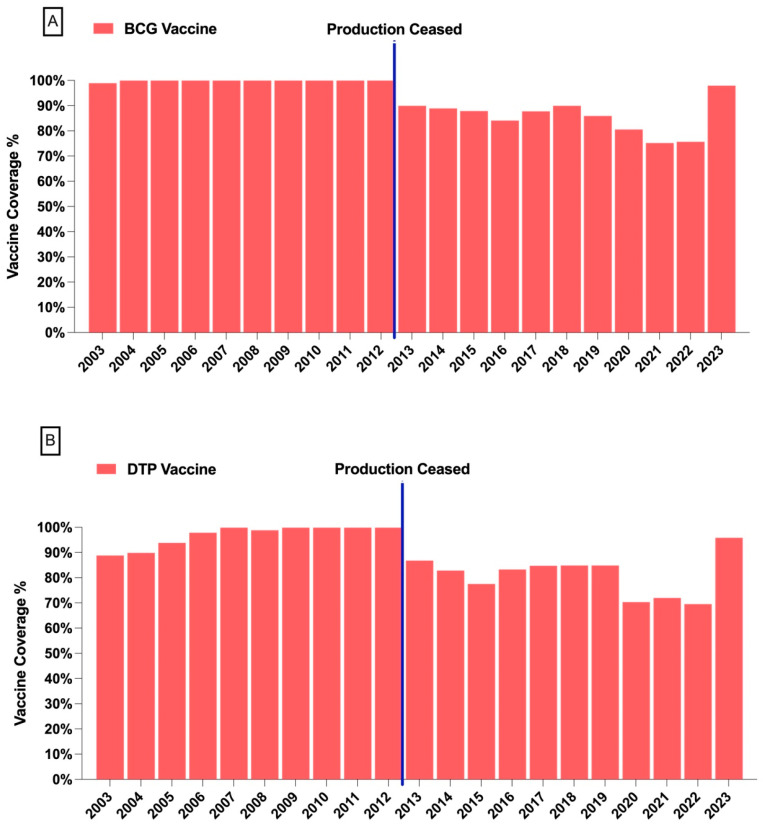
(**A**) Bacillus Calmette–Guérin (BCG) vaccine coverage (%) from 2003 to 2023. A notable decline is observed following the cessation of local vaccine production in 2014, indicated by the vertical blue line. (**B**) Diphtheria, tetanus, and pertussis (DTP) vaccine coverage (%) from 2003 to 2023. Like the BCG vaccine, a reduction in coverage rates is evident after the cessation of local production.

From 2003 to 2023, vaccine coverage in Ecuador demonstrated significant fluctuations, particularly for the BCG and DTP vaccines. BCG vaccine coverage declined from 99% in 2004 to 89% by 2014 and further dropped to 75% by 2021. A similar trend was observed for the Haemophilus influenzae type B (HiB) vaccine, which peaked at 95% in 2007–2008 before declining to 70% by 2020. These declines coincided with the cessation of local vaccine production in 2014. Despite minor recoveries in recent years, such as an increase to 89% for BCG in 2023, these coverage levels remain below their earlier highs. These findings underscore the pressing need for improved vaccine production infrastructure and robust public health systems to restore and sustain high immunization rates (Figure 1).

### 3.2. Comparison of Coverage Before and After Cessation

#### 3.2.1. Bacille Calmette-Guérin (BCG) Vaccine Coverage

**Figure 2:**
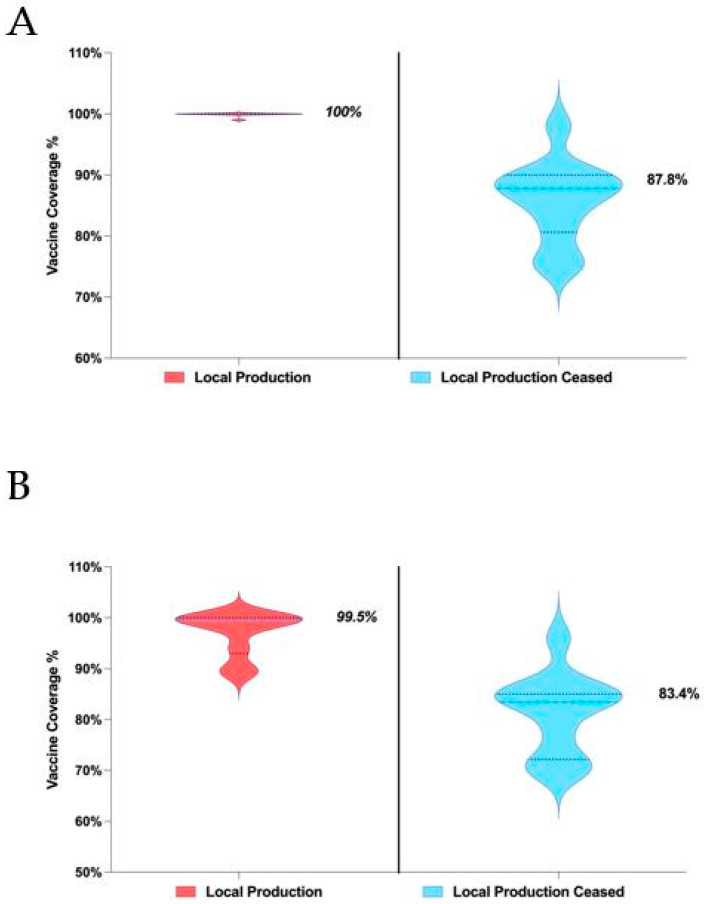
(**A**) Violin plot comparing Bacillus Calmette–Guérin (BCG) vaccine coverage (%) during the Local Production Period (2003–2013) and the Post-Production Cessation Period (2014–2023). Median coverage dropped from 100% during local production to 87.8% after cessation. (**B**) Violin plot comparing diphtheria, tetanus, and pertussis (DTP) vaccine coverage (%) between the Local Production Period (2003–2013) and Post-Production Cessation Period (2014–2023). Median coverage decreased from 99.5% during production to 83.4% post-cessation.

The statistical analysis revealed a significant decline in BCG vaccine coverage following the cessation of local production. The Mann–Whitney U test indicated a highly significant difference (*p* < 0.0001). During the Local Production Period (2003–2013), the median BCG coverage was 100.0%, compared to 87.8% in the Post-Production Cessation Period (2014–2023). The actual median difference was −12.2%, consistent with the Hodges–Lehmann estimate (−12.2%). These findings confirm that the cessation of local production had a substantial impact on BCG coverage (Figure 2).

#### 3.2.2. Diphtheria, Tetanus, and Pertussis (DTP) Vaccine Coverage

A significant reduction was also observed for DTP vaccine coverage. The Mann–Whitney U test indicated a statistically significant decline (*p* < 0.0001). Median DTP coverage during the Local Production Period was 99.5%, while it decreased to 83.4% during the Post-Production Cessation Period. The median difference was −16.06%, supported by the Hodges–Lehmann estimate (−15.06%). This analysis highlights the detrimental effect of ceasing local vaccine production on DTP coverage rates (Figure 2).

#### 3.2.3. Mortality and Incidence Trends in Vaccine-Preventable Diseases

**Figure 3:**
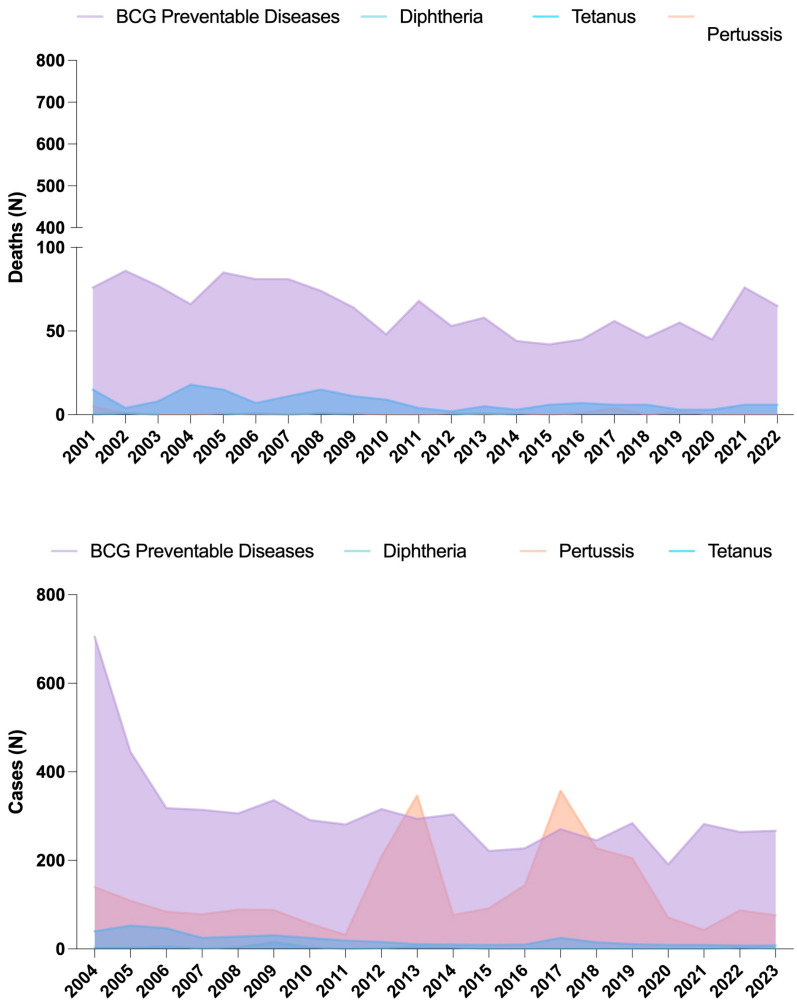
Trends in mortality and incidence of vaccine-preventable diseases in Ecuador (2001–2023).

To assess the impact of local vaccine production cessation in Ecuador (2013) on disease burden, we analyzed mortality and hospital discharge data for BCG-preventable diseases (tuberculosis), tetanus, diphtheria, and pertussis from 2001 to 2023. Mortality trends indicate that while diphtheria-related deaths remained absent post-2013, tuberculosis-related deaths increased from 42 in 2015 to 76 in 2021, suggesting a possible link between declining BCG coverage and increased disease burden. Tetanus-related deaths remained stable, averaging six cases per year post-2013. Pertussis mortality was sporadic before 2013 and absent afterward. Hospital discharge data as a proxy for incidence show a similar pattern, with BCG-preventable disease cases decreasing before 2013 but rising again post-2015, stabilizing around 264–267 cases per year. Pertussis incidence surged in 2013 (347 cases) and peaked in 2017 (358 cases), emphasizing the importance of sustained booster dose administration. These trends highlight the potential consequences of vaccine production interruptions on disease resurgence and underscore the need for stable immunization strategies (Figure 3).

The upper panel depicts the number of deaths related to BCG-preventable diseases (primarily tuberculosis), tetanus, diphtheria, and pertussis from 2001 to 2022. The lower panel illustrates hospital discharge cases, used as a proxy for disease incidence, for the same vaccine-preventable diseases from 2004 to 2023. A notable trend is the increase in tuberculosis-related mortality and incidence after 2013, coinciding with the cessation of local vaccine production. Pertussis cases also peaked in 2013 and 2017, emphasizing the importance of sustained immunization efforts. Diphtheria remained nearly absent, while tetanus cases exhibited minor fluctuations. These trends highlight the potential impact of disruptions in vaccine supply on disease burden.

## 4. Discussion

The findings of this study provide a unique case study highlighting the profound public health challenges linked to the cessation of local vaccine production in Ecuador. The findings show that before 2014, Ecuador had a robust local production infrastructure that supplied critical vaccines, such as Bacille Calmette–Guérin (BCG), diphtheria–tetanus (DT), and diphtheria–tetanus–pertussis (DPT), among others, as shown in Table 1. These locally produced vaccines not only ensured self-sufficiency but also contributed significantly to maintaining high coverage rates, as evidenced by the near-perfect vaccination coverage of 100% for BCG and 99.5% for DTP during the local production period (2003–2013) (Table 1).

However, the cessation of local production in 2014 led to an overreliance on imported vaccines, where 99% of vaccines in Ecuador were sourced from international suppliers after 2014 (Table 2). This reliance introduced supply chain vulnerabilities and coincided with a marked decline in vaccination coverage rates. For instance, BCG coverage dropped to 87.8%, and DTP coverage dropped to 83.4% in the post-production cessation period (2014–2023), with coverage as low as 75% for BCG in 2021. These declines signify a weakened immunization system and increased population vulnerability to preventable diseases.

### 4.1. Importance of Local Vaccine Production: Public Health and Economic Perspectives

Ecuador’s historical production portfolio included vaccines for tuberculosis (BCG), rabies (human and veterinary), and pentavalent vaccines (combining DTP, hepatitis B, and Haemophilus influenzae b), which were essential for safeguarding both human and animal health (Table 1). Local production allowed Ecuador to save on vaccine acquisition costs while maintaining control over supply, ensuring consistent immunization efforts. The loss of this capacity has not only compromised health security but also strained financial resources (Table 2).

As demonstrated, the cessation of local production forced Ecuador to fully rely on imports for critical vaccines, such as MMR (measles, mumps, rubella), rotavirus, pneumococcus, and influenza. For example, the cost of importing pneumococcal conjugate vaccines alone amounted to USD 15.27 million, while pentavalent vaccines cost an additional USD 3.43 million annually. These figures highlight the economic burden of transitioning from local production to complete import dependency, undermining the government’s stated goal of changing the productive matrix toward industrial self-sufficiency.

Despite policy rhetoric about diversifying Ecuador’s economy and promoting industrialization, the closure of vaccine production facilities in 2014 marked a regressive step. The productive matrix failed to shift in the healthcare sector, leaving the country exposed to external supply shocks, as was evident during the COVID-19 pandemic [17]. Local vaccine production would not only reduce import dependency but also generate economic benefits through cost savings and the creation of skilled jobs in biotechnology.

Ecuador’s biotechnological history underscores the importance of sustained governmental and institutional support. As the world shifts towards health resilience and localized production, Ecuador’s historical successes and lessons learned provide a blueprint for rebuilding its vaccine production capacity. Reviving this sector could position Ecuador as a leader in the region, ensuring self-sufficiency and improved public health outcomes.

### 4.2. The Evolution and Unnecessary Closure of Vaccine Production in Ecuador

**Figure 4:**
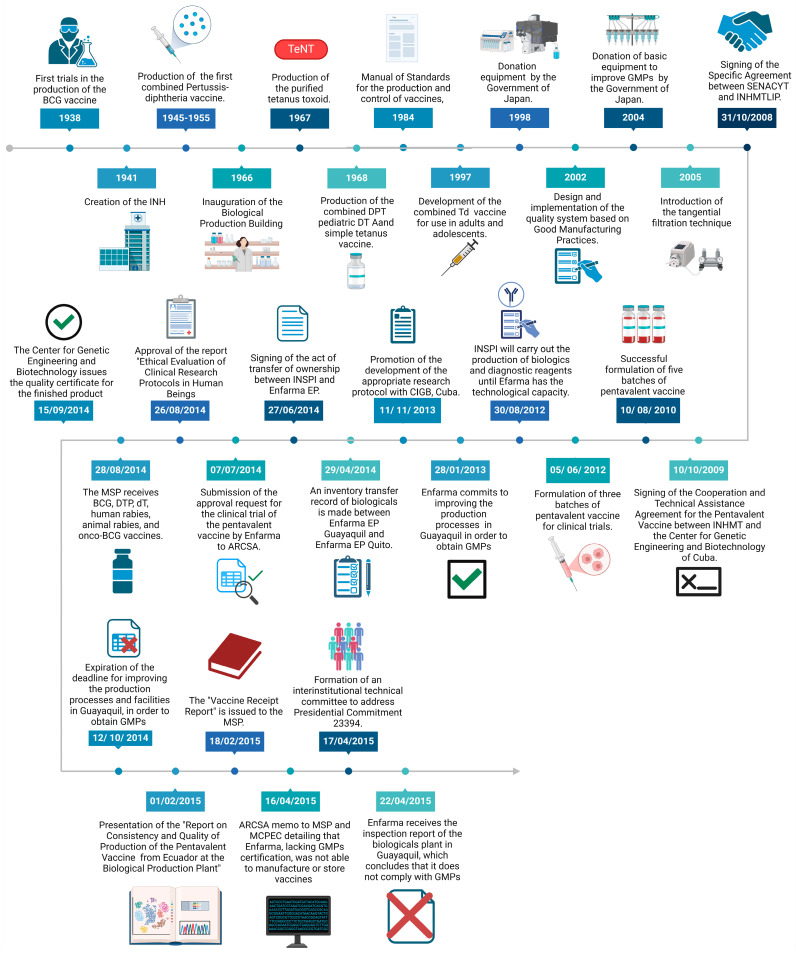
Timeline of the history of vaccine production in Ecuador.

Ecuador’s history of vaccine production reflects a legacy of scientific innovation and public health achievements. Starting in 1938, the country pioneered vaccine production in Latin America, beginning with clinical trials for the Bacille Calmette–Guerin (BCG) vaccine under the leadership of Dr. Pablo Arturo Suárez (Figure 4). In 1941, the creation of the INHMT-LIP marked a significant milestone, establishing an institution dedicated to developing products essential for public health preservation [16].

Throughout the mid-20th century, Ecuador expanded its biotechnological capacity. Between 1945 and 1955, the country successfully produced the pertussis vaccine, purified diphtheria toxoid, and the first combined pertussis–diphtheria vaccine. The 1960s saw further advancements with the production of purified tetanus toxoid in 1967 and the development of combined vaccines, including DPT and DT pediatric formulations, in 1968. These efforts were consolidated with the inauguration of a dedicated biological production facility in 1966 [16].

Ecuador’s commitment to quality was evident in 1984, when the country implemented its first manual for vaccine production and control based on PAHO and WHO standards. By 1997, Ecuador had developed its adult Td (tetanus–diphtheria) vaccine, further enhancing its immunization program [16].

International collaborations also played a crucial role in bolstering Ecuador’s production capacity. In 1998, Japan donated critical equipment to modernize the INHMT-LIP facilities, laying the groundwork for compliance with Good Manufacturing Practices (GMPs). This support continued in 2004 with additional donations that significantly improved the quality and scalability of vaccine production, introducing advanced technologies such as tangential flow filtration for toxoid purification [16].

### 4.3. The Pentavalent Vaccine Initiative and Challenges

One of Ecuador’s most ambitious projects was the development of the pentavalent vaccine (DPT-HB-Hib). In 2009, a cooperation agreement with the Cuban Center for Genetic Engineering and Biotechnology (CIGB) led to the successful formulation of five batches (150,000 doses) of the pentavalent vaccine in 2010. By 2012, three additional batches were prepared for clinical trials. However, disagreements regarding institutional oversight stalled progress. The 2012 presidential decree (Decree 1290) transferred vaccine production responsibilities to Enfarma EP, while requiring compliance with GMP standards [16].

Despite initial optimism, the transition was marred by political disputes and bureaucratic inefficiencies. The MSP imposed stringent GMP requirements that were impractical for existing facilities to meet immediately. These regulations, while essential for long-term compliance, failed to account for the operational realities of the Guayaquil plant, which had been producing effective vaccines for decades. For instance, structural limitations, such as ceiling heights, made it impossible to meet certain GMP criteria without constructing a new facility [16].

### 4.4. The Decline and Closure

In 2014, vaccine production ceased entirely. The MSP and Enfarma EP failed to agree on a strategy to maintain production while upgrading facilities. Promises to construct a new state-of-the-art plant were unfulfilled, and Enfarma EP was held to standards that ignored the historical efficacy and safety of Ecuador’s vaccines. As a result, millions of doses that could have safeguarded public health were left unproduced [16].

This abrupt closure represented a significant loss for Ecuador. Once a leader in regional vaccine production, the country became entirely dependent on imported vaccines by 2016. The financial burden on the national immunization program increased, as shown by historical procurement data. For example, while the cost of locally produced vaccines was modest, reliance on imports escalated, consuming over USD 44 million annually by 2013, with 100% dependency by 2016 [16].

### 4.5. Revitalizing Local Production for Public Health and Economic Resilience

Reviving Ecuador’s vaccine production capacity presents a dual opportunity to bolster public health resilience and foster economic growth. As demonstrated by the historical achievements, Ecuador has a legacy of producing critical vaccines such as BCG, DPT, and pentavalent formulations, which contributed to the country’s immunization success (Table 1). However, the cessation of local production in 2014 marked a significant setback, leaving Ecuador reliant on expensive imports (Table 2).

The re-establishment of local manufacturing could address these challenges. Investments in GMP, scalable production facilities, and workforce development are vital to producing vaccines that meet international standards. Partnerships with global entities like the PAHO and WHO could provide technical expertise and financial resources to modernize Ecuador’s facilities [16].

From an economic perspective, restoring local vaccine production would reduce dependency on costly imports, as demonstrated by the financial breakdown in Table 2**,** where Ecuador spent over USD 44 million on imported vaccines in 2013. Redirecting even a fraction of these funds toward domestic production could alleviate fiscal pressures while strengthening healthcare infrastructure. Furthermore, local production would allow vaccines to be tailored to Ecuador’s epidemiological landscape, enabling faster and more efficient responses to outbreaks [16].

### 4.6. Addressing the Broader Implications

The decline in vaccine coverage and the loss of local production underlines the urgent need for a cohesive national strategy to revitalize Ecuador’s biotechnological capabilities. This effort should prioritize establishing clear regulatory frameworks and financial incentives to promote local vaccine production, modernizing existing facilities such as those at the former Instituto Nacional de Higiene and creating scalable production lines to meet national and regional demands. Strengthening international collaborations is essential for securing technology transfer, capacity building, and funding support, while public awareness campaigns must rebuild trust in vaccination programs by highlighting the benefits of locally produced, high-quality vaccines, particularly in addressing vaccine hesitancy. By adopting these measures, Ecuador could emerge as a regional leader in vaccine production, reducing dependence on imports and enhancing health equity and security.

Vaccine production is rarely profitable due to high research, development, and regulatory costs, yet its public health and economic benefits are substantial. The cessation of local production in 2014 weakened health resilience and stalled biotechnological progress, leaving Ecuador dependent on costly imports and vulnerable to supply chain disruptions. Countries with domestic manufacturing, like Brazil and India, have demonstrated that investing in local production enhances economic resilience and sustainable vaccine access.

Reestablishing domestic vaccine production is a strategic necessity, ensuring faster pandemic response, reduced foreign dependency, and tailored immunization strategies. Ecuador’s past successes, including BCG and pentavalent vaccines, highlight its capacity for high-quality manufacturing with targeted investment. Economically, local production could save over USD 40 million annually in imports, generate jobs, and strengthen Ecuador’s position in biotechnological innovation. Addressing infrastructure gaps, workforce training, and regulatory compliance is essential to regaining competitiveness in the field.

Achieving this requires political commitment, sustained investment, and long-term planning. Strengthening regulatory frameworks, fostering public–private partnerships, and leveraging international collaborations are critical steps. Additionally, Ecuador must overcome bureaucratic inefficiencies and institutional fragmentation, which have historically hindered progress. With the right policies, Ecuador can rebuild vaccine manufacturing, enhance public health security, and drive economic growth, positioning itself as a regional leader in biotechnology.

### 4.7. Barriers in Ecuador for Receiving Vaccines

A study that analyzed the causes of non-compliance with vaccination schedules in children under two years old revealed that the main causes were issues with working hours or lack of time, forgetting vaccination dates, previous negative experiences or negative experiences from acquaintances, prohibition by the partner of the caregiver, lack of knowledge about vaccines, long waiting times to access vaccines, and a shortage of vaccines in institutions [18]. During the pandemic, the barriers to receiving vaccines included the belief that vaccines could be unsafe due to potential side effects and misconceptions about the effectiveness and safety of vaccines [19]. It is also important to highlight the presence of geographical barriers in rural areas of Ecuador, where in many cases, the long distances to health centers prevented the population from attending [20,21].

### 4.8. Impact of Declining Vaccination Coverage on Disease Resurgence

The vaccination campaigns most affected have been those for measles, polio, diphtheria, whooping cough, tetanus, and meningitis, which is why some preventable diseases through vaccination are resurging in different parts of the world [22]. In Ecuador, it has been observed that in 2021, the coverage of the polio vaccine did not exceed 50% in 16 cantons of the country. The dropout rate for the pentavalent vaccine, which protects against whooping cough, diphtheria, tetanus, Haemophilus influenzae type B, and hepatitis B, is 21%, 19%, and 18% in the provinces of Orellana, Sucumbíos, and Pichincha, respectively. Other vaccines, such as the human papillomavirus vaccine, which is administered to children under 9 years old, had a coverage rate that did not exceed 28% [23].

## 5. Limitations

This study acknowledges several limitations that may influence the interpretation of its findings. First, it relies on secondary data from official records, which may contain gaps or inconsistencies, particularly for the period prior to 2003. Second, external factors such as global pandemics, economic crises, and policy shifts could have affected vaccination coverage trends, making it challenging to isolate the direct effects of the cessation of local vaccine production. Third, while we included an analysis of disease incidence and mortality trends, we did not perform a formal statistical association between vaccine coverage decline and disease occurrence, which could strengthen the causal inference. Fourth, we did not have access to individual-level vaccination data, making it impossible to directly assess associations between vaccination status, socioeconomic status (SES), or demographic factors. The lack of detailed data on unvaccinated individuals prevents further exploration of potential disparities in vaccine access and uptake. Finally, due to the COVID-19 pandemic, vaccination coverage worldwide decreased considerably. These pandemic years were considered to compare coverage, even though the effects of the pandemic on vaccination coverage were not adjusted.

Despite these limitations, this study provides valuable insights by systematically analyzing the available data. It highlights the critical relationship between local vaccine production and immunization coverage in Ecuador, offering a robust foundation for further research and policy development. Future studies should consider integrating individual-level data and statistical modeling to better understand the broader implications of vaccine production policies on disease burden and health system resilience.

## 6. Conclusions

Ecuador’s history of vaccine production offers a valuable roadmap for rebuilding its biotechnological capabilities. Despite the challenges posed by the cessation of local production, the lessons learned provide a strong foundation for future success. The local production of vaccines is essential to maintain adequate population coverage. Our results highlight the negative impact of halting local vaccine production and relying entirely on imports. This is evident in the case of the BCG vaccine, where coverage dropped from 100% to 87.8%. Our findings emphasize the need to generate health policies that support and incentivize local vaccine production, as well as to foster international collaboration and strategic planning, which can help overcome current challenges and position Ecuador as a regional leader in vaccine production and public health resilience.

## Figures and Tables

**Table 1:** Vaccines produced locally in Ecuador and their target pathogens.

Population	Vaccine	Pathogen
Adults and children	Human rabies vaccine	Rhabdoviridae, genus Lyssavirus type 1
Animals	Veterinary rabies vaccine	Rhabdoviridae, genus Lyssavirus type 1
Adults	Adult DT vaccine	Diphtheria + tetanus
Children	Pediatric DT vaccine	Diphtheria + tetanus
Children	Triple DPT vaccine	Diphtheria + tetanus + pertussis
Children	Pentavalent vaccine	Diphtheria + tetanus + pertussis + hepatitis B + Haemophilus influenzae b
Newborns	BCG vaccine	Tuberculosis
Oncological patients	Onco-BCG vaccine	Superficial bladder cancer

This table outlines the range of vaccines produced domestically in Ecuador before the cessation of local production in 2014. These vaccines served diverse populations, including children, adults, and specific groups such as oncological patients and animals, addressing critical public health needs and zoonotic diseases.

**Table 2:** Cost distribution of vaccines acquired by Ecuador in 2013.

Vaccine Name	Supplier		
	Revolving Fund	Locally Produced	Imported %
BCG	USD 89,647		100%
MMR (Measles, Mumps, Rubella)	USD 852,194		100%
Measles–Rubella	USD 105,344		100%
Polio	USD 402,780		100%
Rotavirus (liquid)	USD 4,011,333		100%
Pneumococcus	USD 819,275		100%
Hepatitis B Immunoglobulin	USD 8807		100%
Varicella	USD 5,129,735		100%
Yellow Fever	USD 1,585,111		100%
Trivalent Seasonal Influenza (Adults)	USD 6,644,748		100%
Trivalent Seasonal Influenza (Pediatric, >3 years)	USD 3,743,625		100%
Trivalent Seasonal Influenza (Pediatric, <3 years)	USD 1,032,000		100%
Pneumococcal Conjugate Vaccine	USD 15,272,250		100%
Adult DT		USD 247,200	0%
Antimeningococcal	USD 29,960		100%
Hepatitis B (Adults)	USD 292,500		100%
Hepatitis B (Pediatric)	USD 25,500		100%
Pentavalent (liquid)	USD 3,431,000		100%
DPT		USD 92,000	0%
Pediatric DT		USD 72,000	0%
Total	USD 43,475,809	USD 164.247	99%

This table highlights the financial allocation for vaccines in Ecuador during 2013, distinguishing between vaccines imported through the revolving fund and locally produced options. Despite the presence of local production capabilities for some vaccines, the overwhelming majority (99%) were imported, illustrating the country’s growing dependency on international suppliers following the cessation of local vaccine production.

## Data Availability

The data that support the findings of this study are available from the corresponding author, (EOP), upon reasonable request.

## Abbreviations

The following abbreviations are used in this manuscript:

LMICs	Low- and middle-income countries
BCG	Bacille Calmette–Guerin
DTP	Diphtheria–tetanus–pertussis
INHMT-LIP	Instituto Nacional de Higiene y Medicina Tropical Leopoldo Izquieta Pérez MSP Ministry of Public Health
PAHO	Pan American Health Organization
WHO	World Health Organization
HiB	Haemophilus influenzae type B
DT	Diphtheria–Tetanus
MMR	Measles, Mumps, Rubella
CIGB	Cuban Center for Genetic Engineering and Biotechnology
